# The Association of Chronic Periodontitis as a Potential Risk Factor with Rheumatoid Arthritis: A Nested Case-Control Study Using a Korean National Health Screening Cohort

**DOI:** 10.3390/biomedicines12050936

**Published:** 2024-04-23

**Authors:** Ho Suk Kang, Joo-Hee Kim, Ji Hee Kim, Woo Jin Bang, Hyo Geun Choi, Dae Myoung Yoo, Na-Eun Lee, Kyeong Min Han, Nan Young Kim, Ha Young Park, Kyueng-Whan Min, Mi Jung Kwon

**Affiliations:** 1 Division of Gastroenterology, Department of Internal Medicine, Hallym University Sacred Heart Hospital, Hallym University College of Medicine, Anyang 14068, Republic of Korea; hskang76@hallym.or.kr; 2 Division of Pulmonary, Allergy, and Critical Care Medicine, Department of Internal Medicine, Hallym University Sacred Heart Hospital, Hallym University College of Medicine, Anyang 14068, Republic of Korea; luxjhee@gmail.com; 3 Department of Neurosurgery, Hallym University Sacred Heart Hospital, Hallym University College of Medicine, Anyang 14068, Republic of Korea; kimjihee.ns@gmail.com; 4 Department of Urology, Hallym University Sacred Heart Hospital, Hallym University College of Medicine, Anyang 14068, Republic of Korea; yybbang@hallym.or.kr; 5 Suseo Seoul E.N.T. Clinic, 10, Bamgogae-ro 1-gil, Gangnam-gu, Seoul 06349, Republic of Korea; mdanalytics@naver.com; 6 Hallym Data Science Laboratory, Hallym University College of Medicine, Anyang 14068, Republic of Korea; ydm1285@naver.com (D.M.Y.); nel2001@hanmail.net (N.-E.L.); hankm1130@naver.com (K.M.H.); 7 Laboratory of Brain and Cognitive Sciences for Convergence Medicine, Hallym University College of Medicine, Anyang 14068, Republic of Korea; 8 Hallym Institute of Translational Genomics and Bioinformatics, Hallym University Medical Center, Anyang 14068, Republic of Korea; honeyny78@gmail.com; 9 Department of Pathology, Busan Paik Hospital, Inje University College of Medicine, Busan 47392, Republic of Korea; hy08.park@gmail.com; 10 Department of Pathology, Uijeongbu Eulji Medical Center, Eulji University School of Medicine, 712, Dongil-ro, Uijeongbu 11759, Republic of Korea; kyueng@gmail.com; 11 Department of Pathology, Hallym University Sacred Heart Hospital, Hallym University College of Medicine, Anyang 14068, Republic of Korea

**Keywords:** chronic periodontitis, rheumatoid arthritis, nested case-control study, national health screening cohort data

## Abstract

Growing research has proposed that rheumatoid arthritis (RA) and chronic periodontitis (CP) share similar pathophysiological mechanisms involving inflammation and tissue destruction. However, the potential correlation of CP as a contributing factor for the occurrence of RA warrants validation in the Korean population, where both diseases are prevalent, especially considering the increasingly aging demographic in Korea. This study examined 5139 RA cases and 509,727 matched controls from a Korean national cohort dataset (2002–2019) by carefully employing propensity score matching to ensure comparability between groups. Baseline characteristics were compared using standardized differences, and logistic regression was employed to estimate the impact of CP history on RA likelihood while controlling for covariates. We fully examined medical records documenting CP occurrences within the two-year period leading up to the index date, conducting comprehensive subgroup analyses. While a 1-year history of CP did not show a significant association with likelihood of RA, a 2-year history of CP increased RA likelihood by 12%, particularly among older adults, females, rural residents, and those with certain comorbidities such as hypercholesterolemia. Interestingly, this association persisted even among individuals with non-smoking habits, normal weight, and infrequent alcohol consumption. These findings suggest that chronic CP exposure for at least 2 years may independently elevate RA risk in Korean adults. The association in certain subgroups appears to suggest a predisposition toward genetic susceptibilities over lifestyle and environmental factors. Predicting RA in CP patients may be challenging, emphasizing the importance of regular RA screening, especially in high-risk subgroups.

## 1. Introduction

Rheumatoid arthritis (RA) is a chronic, systemic inflammatory, and autoimmune disease that poses significant challenges due to its intractable nature, leading to functional disability and early mortality [1,2]. It affects approximately 1% of the global population [2] and exhibits a prevalence ranging from 0.27% to 1.85% in the Korean population specifically [1,3]. While RA can manifest at any age, its incidence notably increases with age, particularly after 50 years [1,2]. Given global aging trends and increased life expectancy, the prevalence of RA is expected to rise [2,4]. In Korea, RA presents a growing public health concern due to the associated higher healthcare costs, increased financial vulnerability, and elevated risk of developing serious comorbidities, as compared to the general population [5]. Prevention strategies, particularly primary prevention, are crucial in mitigating the incidence of RA [6]. The specific etiology of RA remains elusive, although it is known that a complex interplay of genetic and environmental factors contributes to its development [7,8]. These factors include advanced age, female gender, family history of RA, genetic predisposition, smoking, obesity, dietary factors, stress, and chronic inflammation [7,8]. Early and aggressive interventions have demonstrated efficacy in slowing disease progression and reducing long-term disability [6]. Therefore, understanding the risk factors and preclinical pathways leading to clinical RA is essential for effective intervention and management of preclinical RA. 

Chronic periodontitis (CP) is a prevalent inflammatory disorder affecting the supportive structures around the teeth, leading to gradual loss of periodontal tissues and underlying bone [9]. It is a significant contributor to tooth loss globally, impacting around 11.2% of the population [10], with an incidence of 23.4% reported in Korea, making it a common reason for outpatient visits [11]. The new classifications of periodontitis released jointly by the European Federation of Periodontology (EFP) and the American Academy of Periodontology (AAP) in 2018 acknowledge the interplay between periodontal diseases and systemic health, highlighting the impact of risk factors like smoking, diabetes, and genetic predisposition on periodontal health [12]. Recent research has suggested a potential link between CP and the likelihood of developing RA [13,14,15]. CP and RA share pathogenic similarities, including immune responses leading to connective tissue inflammation and subsequent bone destruction [16], as well as common risk factors such as increased age, smoking, alcohol consumption, and obesity [7,14]. The hypothesis that the virulence mechanisms of periodontal pathogens (gram-negative, non-motile, facultative anaerobes) such as *Porphyromonas gingivalis* or *Aggregatibacter actinomycetemcomitans*, along with inflammatory mediators from periodontal lesions, could initiate systemic inflammation and contribute to RA pathogenesis, has been proposed and supported by research findings [17,18,19,20]. Both CP and RA exhibit excessive chronic inflammatory reactions characterized by the infiltration of T and B lymphocytes, neutrophils, and monocytes, alongside an imbalance between pro-inflammatory and anti-inflammatory cytokines [17,18]. Cytokines such as interleukins (IL)-1β, IL-2, IL-6, and tumor necrosis factor (TNF)-α, along with their receptors, are implicated in tissue destruction and bone loss in both conditions [17,18], and are reported to connect periodontitis and other immune-mediated inflammatory diseases [21]. Notably, periodontal pathogens such as *Porphyromonas gingivalis* and *Aggregatibacter actinomycetemcomitans* may release a specific enzyme called peptidyl arginine deaminase, which induces protein citrullination and may subsequently trigger infection-driven activation of macrophages and B cells [19,20,22]. This process may mimic the formation of anti-citrullinated protein antibodies, leading to a citrulline-specific autoimmune response, which is unique to RA and implicated in RA development [19]. Citrullinated proteins and related antibodies have been detected in the blood and joints of RA patients, as well as in the inflamed gingival tissue of CP patients [19,23,24], indicating potential interplay between oral infections and RA pathogenesis and highlighting a direct biological connection between CP and RA. 

However, the nature of the association of CP as a contributing factor in the development of RA remains controversial, with conflicting evidence having been obtained from various epidemiological studies across different global regions. In two large-scale prospective cohort studies based on the US population, discrepant results were observed [25,26]. However, over the past two decades of cohort studies in the field, slightly more studies (60%) have favored statistical significance regarding the association between periodontitis and the onset of RA [27,28,29]. Among Asian studies, three Taiwanese nationwide population-based studies have revealed a notable association between a history of periodontitis and subsequent development of RA [27,28,29]. Nevertheless, as these studies could not definitively ascertain whether RA might have developed before the last periodontitis-related visit, especially when the lag time was less than three months [27,28,29], they presented a potential limitation regarding the inclusion of patients with pre-existing RA. Additionally, two nationwide epidemiological studies focusing on the impact of RA on the occurrence of periodontal disease among Korean individuals were identified [30,31], but these diverge from the purpose of our study. These studies solely examined the prevalence of periodontal disease among patients with RA [30,31]. Thus, whether a history of CP may influence the probability of developing RA among the general population, based on the duration of CP history, remains unknown. While some studies have reported more severe periodontal conditions in RA patients compared to healthy controls [32,33], the impact of chronic exposure to CP has not been thoroughly explored.

In this study, our aim was to elucidate the relationship between a previous history of CP and the incident occurrence of RA. We hypothesized that certain risk factors associated with CP history could predict the development of RA. Building upon earlier Korean research [30,31], we investigated the relationship between prior CP history and incident RA development, considering the frequency and duration of CP episodes, as well as associated risk factors predictive of RA incidence. To investigate this, we conducted a nationwide cohort study utilizing a precisely matched nested case-control design, utilizing a comprehensive database spanning almost two decades. We also conducted thorough subgroup analyses to assess the potential effects of CP on the onset of RA, taking into account different timeframes and adjusting for relevant confounders. Additionally, we implemented a 2-year washout period to exclude pre-existing RA patients from our analysis.

## 2. Materials and Methods

### 2.1. Research Methodology and Subjects 

This study was approved by the Ethics Committee (IRB No. 2019-10-023), and written informed consent was waived given the secondary analysis of anonymized retrospective data. 

This retrospective cohort study utilized the Korean National Health Insurance Service-Health Screening Cohort (KNHIS-HSC) database, providing the anonymized electronic health records of a representative sample of the Korean population for research, while ensuring individual privacy through identification prevention measures [34]. South Korea’s National Health Insurance Service covers over 98% of the population through a mandatory policy, ensuring broad representation. In this study, diagnostic coding followed the International Classification of Diseases, 10th Revision, Clinical Modification (ICD-10-CM). RA patients were identified from medical claim and prescription codes. Initially, 5139 RA-affected participants were selected from 514,866 adults aged 40 years and older, spanning 895,300,177 medical claim codes from 2002 through 2019. To address the potential bias from pre-existing RA and remove insidious cases [35], we implemented a “2-year washout period” (*n* = 1570), excluding diagnosed diseases within the initial 2 years (2002 and 2003) at the start of the study, alongside individuals lacking fasting blood glucose data (*n* = 1).

The control cohort, consisting of individuals who had never been diagnosed with RA, was retrieved from the KNHIS-HSC database spanning from 2002 to 2019 (*n* = 509,727). Random number ordering was applied to reduce potential selection bias. Control members were excluded if they had a probable RA diagnostic code and/or a history of RA-related medication at any time during the study period (*n* = 86,934).

**Figure 1:**
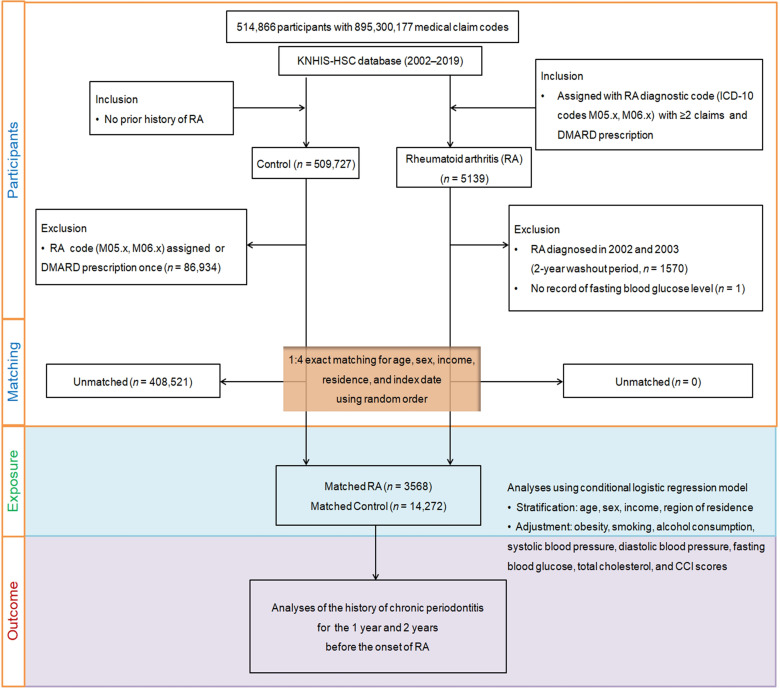
Diagram illustrating the stepwise participant selection process employed in the study. Beginning with the initial cohort of 514,866 individuals sourced from the Korean National Health Insurance Service-Health Screening Cohort (KNHIS-HSC) database, a meticulous selection process culminated in the matching of 3568 rheumatoid arthritis (RA) patients with 14,272 control subjects via propensity score matching, which considered factors such as age, sex, income, and residential area.

To optimize the balance of baseline characteristics between the RA and control groups, propensity score matching was conducted based on age, sex, income, and residence. Random clustered sampling was employed to further mitigate possible selection bias. Each RA patient was individually matched with control participants based on similar propensity score values. The index date for RA patients was defined as the day of electronic assignment of ICD-10 codes for RA in the health insurance datasets. Control participants shared index dates with their matched RA patients, ensuring each pair had the same reference date. These stringent matching procedures resulted in the removal of 408,521 unmatched control members, leaving 3568 individuals with RA successfully paired with 14,272 control participants in a 1:4 ratio for analysis. The study then assessed both groups for CP occurrences within 1-year and 2-year intervals before the index date (Figure 1).

### 2.2. Rheumatoid Arthritis (Outcome)

RA was defined by the presence of at least one corresponding ICD-10 code (M05 or M06) assigned after more than two clinic visits and a prescription for a biological agent or any disease-modifying anti-rheumatic drugs (DMARDs) under the RA diagnostic code [36]. This diagnostic approach in a claim database has demonstrated high sensitivity and accuracy rates in previous studies [37]. 

### 2.3. Chronic Periodontitis (Exposure)

To ensure accuracy and minimize false positives, only individuals treated for CP by dentists with a confirmed diagnosis using the specific ICD-10 code (K05.3) were included [38]. The frequency of clinic or hospital visits related to CP treatment was recorded annually and aggregated over a two-year period before the index date to provide a comprehensive representation of treatment patterns. This meticulous approach aimed to strengthen the relationship between CP and the investigated outcomes [39]. 

### 2.4. Covariates

Demographic information and health data, including age, income, habitation area, smoking history, alcohol consumption, body mass index (BMI), blood pressure, fasting blood glucose, and total cholesterol levels, were collected and recorded. Age groups were categorized into five-year intervals, income into five classes, and habitation areas into urban (representing the seven largest cities in Korea) or rural (encompassing the remaining regions). Similarly, smoking history (non-smoker, former smoker, current smoker), alcohol consumption (<1 time per week, ≥1 time per week), and BMI were categorized accordingly [40]. The BMI categories included underweight (<18.5 kg/m^2^), normal weight (≥18.5 to <23 kg/m^2^), overweight (≥23 to <25 kg/m^2^), obese I (≥25 to <30 kg/m^2^), and obese II (≥30 kg/m^2^) [41]. The Charlson Comorbidity Index (CCI), a widely used tool for assessing the burden of medical conditions, was calculated based on 17 potential comorbidities, including diabetes, renal disease, cardiovascular diseases, pulmonary disease, connective tissue disorders, peptic ulcer, liver disease, cancer, and human immunodeficiency virus infection [42].

### 2.5. Statistical Analyses

To address confounding factors and mitigate selection bias, we employed propensity score matching [43]. Propensity scores were calculated using multivariable logistic regression with baseline covariates. A nearest-neighbor matching algorithm paired patients with RA and control participants based on closest propensity scores [43]. 

We used standardized differences to compare general characteristics between groups, as traditional statistical tests might have yielded false positives, given the large sample sizes [44]. Balance between groups was assessed by examining absolute standardized differences of covariates before and after matching, with values of ≤0.20 indicating good balance [43]. For covariates with standardized differences exceeding 0.20 after matching, additional adjustments were made using multivariable logistic regression analysis [43]. Categorical data were presented as numbers and percentages, while continuous data were reported as means and standard deviations.

The study investigating the relationship between CP (exposure) and RA (outcome) utilized conditional logistic regression models to analyze odds ratios (ORs) and 95% confidence intervals (CIs). Three models were employed: a crude model controlling for age, sex, income, and residence; an adjusted model (model one) accounting for potential confounders such as smoking, alcohol consumption, obesity, and CCI scores; and a further adjusted model (model two) incorporating additional information such as fasting blood glucose, total cholesterol, and blood pressure. CP histories were classified according to treatment frequency (≥1, ≥2, and ≥3 within 1 year, and ≥1 within 2 years), and subgroup analyses were performed with all covariates considered for each CP history. Statistical analysis was carried out using SAS version 9.4 (SAS Institute Inc., Cary, NC, USA), adopting a significance threshold where a two-tailed *p*-value less than 0.05 indicated statistical significance.

## 3. Results

### 3.1. Baseline Demographics

Our study included 5139 individuals diagnosed with RA and 509,727 matched control participants, drawn from a comprehensive database spanning from 2002 to 2019. Both groups were meticulously matched based on age, sex, economic status, and geographical location, resulting in a standardized difference of 0.00, indicating perfect demographic alignment. Other baseline demographic and clinical characteristics exhibited standardized differences of ≤0.20, indicating minimal disparities. However, the CCI score revealed a borderline significant difference between the groups, with a standardized difference of 0.21 (Table 1).

### 3.2. RA Odds Ratios in Relation to CP Histories

To ensure the robustness of our findings, we conducted a comprehensive analysis, examining occurrences of CP within both 1-year and 2-year periods preceding the index date. 

We did not observe any significant differences in the ORs for developing RA between the RA group and controls when considering ≥1 CP history within a 1-year timeframe before the index date. This lack of significant difference between the two groups persisted consistently for participants with CP ≥ 2 or CP ≥ 3 histories within 1 year.

On the other hand, when evaluating the occurrence of at least one instance of CP history within a two-year timeframe preceding the index date, we observed an OR of 1.12 (95% CI = 1.04–1.21, *p* = 0.005) after comprehensive adjustment, signifying a 12% heightened likelihood of RA (Table 2).

### 3.3. Subgroup Analysis

Regarding thorough subgroup analyses categorizing patients based on various parameters, for individuals with at least one CP history within a one-year timeframe before the index date, a subgroup comprising rural residents and those with a CCI score of 0 exhibited a slightly increased likelihood of developing RA. However, these associations showed inconsistency and transience across different subsets. Collectively, our analyses revealed no statistically significant association between any previous history of CP within 1 year before the onset of RA (Supplementary Tables S1–S3).

**Figure 2:**
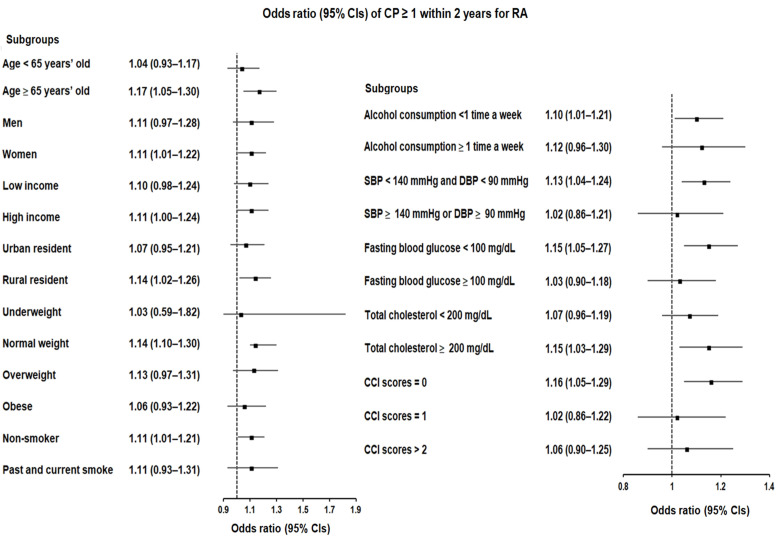
Forest plots illustrating the adjusted odds ratio and corresponding 95% confidence intervals (CIs) for demographic, lifestyle, and comorbid factors related to chronic periodontitis (CP) concerning the development of rheumatoid arthritis (RA) when individuals had been diagnosed with CP ≥ 1 within 2 years preceding the index date.

On the other hand, there was a significant association with at least one instance of CP history within a 2-year timeframe preceding the index date, which persisted notably among participants aged 65 years or older, females, rural residents, non-smokers, individuals with normal weight or total cholesterol levels ≥200 mg/dL, those who consumed alcohol less than once a week, those who had a CCI score of 0, and individuals with no hypertension or hyperglycemia (Figure 2; Supplementary Table S4).

## 4. Discussion

This study aimed to assess the relationship between CP and the likelihood of subsequent RA using a comprehensive and well-matched national cohort dataset encompassing the adult population of Korea. By leveraging propensity matching for demographic factors, conditional logistic regression analysis that considered comprehensive confounding factors, subgroup analyses, and detailed and intensive analysis of medical records of CP history throughout a two-year timeframe, our current investigation found no statistically significant differences in the likelihood of RA development between the study group and controls when examining any occurrences of CP within a 1-year timeframe. Conversely, when extending our analysis to a 2-year period, we noted a 12% higher likelihood of developing RA (95% CI = 1.04–1.21). This effect remained even after adjusting for various covariates, including demographic, socio-economic, and lifestyle factors, and comorbid conditions, all of which may confound both CP and RA. These results suggest that chronic exposure to CP for at least a 2-year period could potentially serve as an independent risk factor for the onset of RA in the adult population of Korea.

Our study supports previous epidemiologic research that has indicated a connection between CP and the risk of developing RA [25,27,28]. A long-term follow-up study of 138 RA patients from the non-institutionalized US adult population over 20 years found that individuals with periodontal disease or more than five missing teeth had higher odds of prevalent and incident RA [25]. To date, around fifteen studies have investigated the association between periodontitis and RA, with nine indicating a link between periodontitis and heightened RA risk, 60% of which demonstrated statistical significance. The most recent meta-analysis in this area revealed that CP patients had a 69% greater risk for RA compared to control individuals [13]. Two prior Korean population-based studies examined the impact of RA as a contributing factor to the occurrence of CP [30,31], which contrasts with the focus of our study. Our study extensively explored the potential connection between prior CP history and RA development among Korean adults. Moreover, we employed a methodologically preferred research design utilizing nationwide organized data, and adjusted for potential confounders. The conclusion of an increased likelihood of RA following CP history was reaffirmed through analysis of a large cohort comprising 3568 RA patients, meticulously matched with 14,272 non-RA participants in respective groups. 

However, conflicting evidence exists regarding the association between CP and RA development. In a prospective Nurses’ Health Study conducted in the US with a cohort of 81,132 participants over 12 years of follow-up, no significant association was found between a history of periodontal surgery and/or tooth loss and an increased risk of developing RA [26]. This cohort study may have been subject to limitations due to its exclusive enrollment of female healthcare professionals, potentially introducing selection bias [26]. Moreover, the definition of exposure to periodontal disease relied on self-reported histories of periodontal surgery or tooth loss within the preceding two years, lacking validation by confirmation of physician’s diagnosis [26], which could introduce recall bias or misclassification due to inaccurate reporting. Additionally, the small number of women with incident RA (only *n* = 33) may have limited the study’s power to detect underlying associations [25,26].

We observed a 12% heightened likelihood of RA when extending our analysis to a 2-year period, whereas we observed a lack of significant differences in the likelihood for developing RA between the study group and controls when repeatedly considering any history of CP within a 1-year timeframe. The lack of significant association between CP and RA within a 1-year timeframe before the index date suggests that the immediate or short-term effects of periodontal inflammation may not play a substantial role in triggering RA onset. However, our findings regarding a 12% increased likelihood of RA occurrence within a 2-year timeframe following CP history highlight a potential longer-term influence of periodontal inflammation on RA development. Similarly, a Taiwanese case-control study also demonstrated that the association between a history of periodontitis and the likelihood of RA is time-dependent, exhibiting a dose-response pattern [27]. A prior study revealed that individuals with severe CP showed notably elevated mean disease activity of RA, which was observed over a 2-year period in 56 patients diagnosed with RA [33]. 

In the present study, subgroup analyses revealed transient associations between CP and the occurrence of RA among certain subsets with specific clinical characteristics, including older adults aged 65 or above, females, rural residents, and those with certain comorbidities like hypercholesterolemia. These subgroups exhibited a more notable increase in the likelihood of developing RA among those with a prolonged history of CP over a 2-year period. Interestingly, this association persisted even among individuals with non-smoking habits, normal weight, and infrequent alcohol consumption, and individuals without hypertension or hyperglycemia. These findings suggest that predicting RA in patients with CP may be challenging and complex, highlighting the need for regular screening of RA in these corresponding patients. Earlier research has similarly indicated that older age, female sex, and non-smoking status exerted significant influence in this correlation [25,33,45]. In particular, a longitudinal study conducted within the US population revealed that females with periodontal disease exhibited higher odds of developing RA, with an OR of 1.47 [25], a figure comparable to our study’s OR of 1.11 (95% CI = 1.01–1.22). Additionally, older age was associated with increased odds of incident RA in their study, with an OR of 1.10 [25], mirroring our finding of an OR of 1.17 (95% CI = 1.05–1.30) in the subgroup aged ≥65 years in the CP group. Another longitudinal analysis based on a US community cohort also indicated an elevated incidence of RA among non-smoking patients with moderate to severe periodontitis [46], aligning with our observation of a correlation between non-smokers and chronic CP exposure. Although environmental risk factors, such as smoking, socioeconomic status, obesity, and hyperglycemic status may influence the both risk of RA and CP [7], here we found significant associations between non-smoking status, normal weight, no comorbidities, and low alcohol consumption with increased likelihood of RA among chronic CP patients followed up after 2 years. Chen et al. [27] reported a correlation between geographical regions and the risk of RA among periodontitis patients, aligning with our finding that rural residents with chronic CP had a higher likelihood of RA. Additionally, the authors identified a decreased RA risk among CP patients with diabetes [27], which seems consistent with our results that showed that individuals without hyperglycemia had a more pronounced increase in the likelihood of developing RA. It is plausible to hypothesize that smoking components or hyperglycemia could potentially disrupt the production of cytokines or inflammatory mediators, resulting in the imbalance in cytokine production observed among CP patients who smoke or have hyperglycemia [47,48], and potentially leading to unexpected paradoxical associations. Indeed, in a Swedish population-based study, smokers exhibited the lowest levels of interferon (IFN)-γ among RA patients with periodontitis [24]. While one population-based study has reported an association between a history of periodontitis and increased RA risk in newly-treated diabetic patients [29], it did not consider factors such as smoking, alcohol consumption, or other comorbidities in the analysis. Additionally, the study had a small number of incident RA cases, which may have limited the number of covariates for adjustment and potentially introduced confounders [29]. Taken together, these paradoxical associations suggest a complex interplay between environmental factors, socioeconomic status, and individual predisposition in the pathogenesis of RA in CP patients [7,8]. It is possible that a subset of the associations between CP and incident RA development may be more influenced by relevant genetic susceptibilities, rather than lifestyle or environmental factors [8,49].

Several plausible causal mechanisms have been proposed to explain the underlying relationship between CP and new-onset RA. Both RA and CP are characterized as multifactorial diseases and involve sustained chronic inflammation, sharing similarities in adaptive immune phenotype, imbalances between pro- and anti-inflammatory cytokines, and common risk factors and genetic backgrounds [16]. RA and CP exhibit similar cytokine profiles, with elevated levels of pro-inflammatory cytokines (IL-1β, IL-2, IL-6, and TNF-α) [17,18]. Further studies evaluating cytokine gene polymorphisms encoding IL-1, IL-2, IL-4, IL-6, IL-10, TNF-α, and transforming growth factor-β 1, along with a shared genetic background of IL-1, have supported these observations [50]. Since CP represents dysbiotic disease [51], specific periodontal bacteria like *Porphyromonas gingivalis* and *Aggregatibacter actinomycetemcomitans* may contribute to RA autoantibody production, including anti-citrullinated protein antibodies, either through direct post-translational modification of proteins or indirectly by influencing neutrophil-mediated neo-epitope generation [14,52]. The antibodies, which have been demonstrated to be almost exclusive to RA patients, can be detected very early in the disease and are indicative of the clinical outcome of RA [53]. Originating locally in the inflamed synovium, they suggest a potential involvement in the disease process of RA [53]. Additionally, *Porphyromonas gingivalis* and *Treponema denticola* share numerous peptide sequences with epitopes derived from RA-related autoantibodies, such as RA-A47, creating potential cross-reactivity [54]. These correlations may result in bacterially induced immunologic dysregulation, leading to RA development, especially in genetically susceptible individuals (such as those positive for the shared epitope). Indeed, since the genetic contribution to RA and periodontitis is substantial and may account for 60% and 50% of the RA and CP risk profile, respectively [55,56], some genetic variations, particularly the HLA-DRB1 alleles associated with shared epitope coding, may enhance susceptibility to RA development in CP patients, as both diseases share genetic risk factors like MHC class II HLA-DRB1 alleles and cytokine single nucleotide polymorphisms, including the *KCNQ1* gene [57]. In addition, the A allele of rs2430561 in the *IFNγ* gene, and the T allele of rs2476601 in the *PTPN22* gene, have been found to be significantly associated with patients who have comorbidities of both RA and CP [58,59]. A connection between anti-citrullinated protein antibodies and IL-10 polymorphisms has been discovered, indicating that IL-10 expression may confer protection against periodontal disease when anti-citrullinated protein antibodies are present in the bloodstream [55]. Notably, males have been shown to predominantly exhibit IL-10 predisposing genes, whereas females show a higher propensity for the expression of genes associated with rheumatoid factor and anti-citrullinated protein antibody [55]. This sex-dependent genetic correlation suggests that females with CP may be at a higher risk of developing RA compared to males in our study. 

A Mendelian randomization study has also provided weak yet significant evidence suggesting a causal association between periodontitis and the occurrence of RA [15]. However, two other Mendelian randomization studies did not find evidence that genetic susceptibility to periodontitis is linked to the occurrence of RA [60,61]. These studies focused exclusively on individuals of European descent with both periodontitis and RA [15,60,61]. Importantly, the samples included individuals with established or chronic RA [60]; this differs from pre-clinical RA candidates or individuals at risk of RA, which is the target group in our study. This may warrant caution in interpretation, and underscores the need for verification of the relationship between these two diseases in Asian populations.

While our study may contribute to supporting the growing body of epidemiologic evidence on the link between CP and RA, several limitations must be acknowledged. Firstly, given the observational and retrospective nature of the study design, it cannot definitively establish a causal relationship between CP and the development of PD. Secondly, our research primarily relies on administrative data and does not delve into potential biological mechanisms linking CP and PD, leaving a gap in our understanding of the underlying pathophysiology. This may also introduce biases and limitations in relation to accurately capturing disease diagnoses and clinical parameters. Third, our study focuses on CP histories within specific time frames (1-year and 2-year periods), which may not capture the potential longer-term effects of CP on RA development over the observed time frames. Nonetheless, the previous two long-term population-based studies did not provide consistent findings regarding the association between the two diseases. A 20-year US study on RA patients revealed higher odds of prevalent and incident RA among individuals with periodontal disease or more than five missing teeth [25]. However, a separate 12-year US study found no significant association between periodontal surgery/tooth loss history and increased RA risk [26]. Additionally, our findings are based on a specific dataset derived from Korean population data of adults aged 40 and older. Consequently, the relevance of our results, when generalized to different ethnic populations or demographic settings, may be limited. While our study identified a slightly increased likelihood of RA in certain subgroups, it is important to note that these findings were modest and may warrant further investigation to determine their clinical significance. Moreover, the dataset used, the KNHIS-HSC database, lacked detailed information on factors such as severity grade of CP and RA, family medical history, personal genetics, and dietary habits. Unfortunately, this study did not incorporate the treatment outcomes of CP, and the deadline for KNHIS-HSC data accessibility has now expired. Patients with RA and comorbid CP who undergo non-surgical periodontal treatment have demonstrated improvements in clinical outcomes for RA [62]. However, the consistency of the treatment effect appears to be variable [63]. Consequently, we were unable to assess whether the remission of CP following successful treatment has any impact on the development of RA. This omission represents another potential bias that should be taken into consideration. Our analysis did not account for these missing details. Therefore, despite efforts to adjust for known confounding factors, there is always the possibility of lingering confounders due to undisclosed or unrecorded residual variables that could influence study outcomes. These limitations may constrain our ability to account for possible undisclosed variables as confounding factors.

Nonetheless, this study boasts notable strengths that enhance its robustness. One key strength lies in the utilization of a substantial and representative sample from the Korean adult population, comprising a significant number of participants—3568 RA patients and 14,272 well-matched controls—carefully gathered from a validated nationwide healthcare database. This extensive sample instills confidence in the study’s outcomes. Additionally, the meticulous matching of groups based on demographic characteristics, the careful attention given to the analysis timeframe, and the rigorous adjustment performed for confounding variables further bolster the reliability of the findings. To mitigate the possibility of bias from pre-existing RA and eliminate insidious cases, we excluded diagnosed diseases within the first two years at the start of the study (*n* = 1570). We also analyzed full medical records documenting CP occurrences within the two-year period leading up to the index date. This methodological approach minimizes the impact of potential confounding factors as much as possible. Moreover, the study’s detailed subgroup analysis, incorporating variables such as demographic, lifestyle, and medical factors, provides a nuanced understanding of the relationship between CP and RA across diverse population segments. The comprehensive nature of the KNHIS-HSC dataset, encompassing complete medical histories from across the entire country, enhances the study’s generalizability and precision. This breadth allows for a more thorough exploration of the potential link between CP and RA. 

## 5. Conclusions

In conclusion, our study supports a positive association between a history of chronic CP and the subsequent likelihood of developing RA in the adult Korean population. Subgroup analyses unveiled transient associations between CP and RA occurrence among certain subsets with specific clinical characteristics, underscoring the complexity of predicting RA in CP patients. These findings may emphasize the potential importance of regular RA screening in chronic CP patients with a history of at least 2 years. Further research is crucial to explore the longer-term implications of CP on RA, and to elucidate the biological mechanisms underlying this relationship.

## Figures and Tables

**Table 1:** A summary of the demographic and clinical characteristics for this study.

Characteristics	Total Participants		
	RA	Control	Standardized Difference
Age (y), *n* (%)			0.00
40–44	63 (1.77)	252 (1.77)	
45–49	333 (9.33)	1332 (9.33)	
50–54	654 (18.33)	2616 (18.33)	
55–59	706 (19.79)	2824 (19.79)	
60–64	660 (18.50)	2640 (18.50)	
65–69	514 (14.41)	2056 (14.41)	
70–74	343 (9.61)	1372 (9.61)	
75–79	213 (5.97)	852 (5.97)	
80–84	68 (1.91)	272 (1.91)	
85+	14 (0.39)	56 (0.39)	
Sex, *n* (%)			0.00
Male	1055 (29.57)	4220 (29.57)	
Female	2513 (70.43)	10,052 (70.43)	
Income, *n* (%)			0.00
1 (lowest)	611 (17.12)	2444 (17.12)	
2	530 (14.85)	2120 (14.85)	
3	585 (16.40)	2340 (16.40)	
4	747 (20.94)	2988 (20.94)	
5 (highest)	1095 (30.69)	4380 (30.69)	
Region of residence, *n* (%)			0.00
Urban	1541 (43.19)	6164 (43.19)	
Rural	2027 (56.81)	8108 (56.81)	
Obesity, *n* (%)			0.04
Underweight	69 (1.93)	319 (2.24)	
Normal	1334 (37.39)	5134 (35.97)	
Overweight	945 (26.49)	3862 (27.06)	
Obese I	1115 (31.25)	4476 (31.36)	
Obese II	105 (2.94)	481 (3.37)	
Smoking status, *n* (%)			0.03
Nonsmoker	2853 (79.96)	11,551 (80.93)	
Past smoker	308 (8.63)	1204 (8.44)	
Current smoker	407 (11.41)	1517 (10.63)	
Alcohol consumption, *n* (%)			0.04
<1 time a week	2690 (75.39)	10,492 (73.51)	
≥1 time a week	878 (24.61)	3780 (26.49)	
Systolic blood pressure (*n*, %)			0.05
<120 mmHg	1161 (32.54)	4675 (32.76)	
120–139 mmHg	1764 (49.44)	6645 (46.56)	
≥140 mmHg	643 (18.02)	2952 (20.68)	
Diastolic blood pressure (*n*, %)			0.04
<80 mmHg	1826 (51.18)	6977 (48.89)	
80–89 mmHg	1211 (33.94)	4928 (34.53)	
≥90 mmHg	531 (14.88)	2367 (16.58)	
Fasting blood glucose (*n*, %)			0.07
<100 mg/dL	2381 (66.73)	9154 (64.14)	
100–125 mg/dL	925 (25.92)	3878 (27.17)	
≥126 mg/dL	262 (7.34)	1240 (8.69)	
Total cholesterol (*n*, %)			0.04
<200 mg/dL	1907 (53.45)	7302 (51.16)	
200–239 mg/dL	1178 (33.02)	4848 (33.97)	
≥240 mg/dL	483 (13.54)	2122 (14.87)	
CCI score (*n*, %)			0.21
0	1991 (55.80)	9045 (63.38)	
1	740 (20.74)	2298 (16.10)	
≥2	837 (23.46)	2929 (20.52)	
The number of CP treatments (Mean, SD)			
within 1 year	0.50 (1.31)	0.49 (1.31)	0.04
within 2 years	0.94 (1.98)	0.93 (2.07)	0.05

Abbreviations: RA, rheumatoid arthritis; CCI, Charlson Comorbidity Index; SD, standard deviation; CP, chronic periodontitis.

**Table 2:** Crude and adjusted odds ratios of chronic periodontitis (CP) for rheumatoid arthritis (RA) when each group is diagnosed with CP ≥ 1, ≥2, and ≥3 within 1 year, and with CP ≥ 1 within 2 years before the index date.

	RA	Control	Odd Ratios for RA (95% Confidence Interval)					
	(Exposure/Total, %)	(Exposure/Total, %)	Crude †	*p*	Model 1 †‡	*p*	Model 2 †§	*p*
From the index date to the before the 1-year period								
Total participants (*n* = 17,840)								
No CP	2770/3568 (77.6%)	11,219/14,272 (78.6%)	1		1		1	
CP ≥ 1	798/3568 (22.4%)	3053/14,272 (21.4%)	1.06 (0.97–1.16)	0.201	1.08 (0.99–1.19)	0.079	1.09 (0.99–1.19)	0.074
CP < 2	2770/3568 (77.6%)	11,219/14,272 (78.6%)	1		1		1	
CP ≥ 2	798/3568 (22.4%)	3053/14,272 (21.4%)	1.04 (0.93–1.17)	0.501	1.07 (0.95–1.20)	0.283	1.07 (0.95–1.20)	0.300
CP < 3	3348/3568 (93.8%)	13,401/14,272 (93.9%)	1		1		1	
CP ≥ 3	220/3568 (6.2%)	871/14,272 (6.1%)	1.01 (0.87–1.18)	0.888	1.04 (0.89–1.21)	0.612	1.04 (0.89–1.21)	0.625
From the index date to the before the 2-year period								
Total participants (*n* = 17,840)								
No CP	2316/3568 (64.9%)	9529/14,272 (66.8%)	1		1		1	
CP ≥ 1	1252/3568 (35.1%)	4743/14,272 (33.2%)	1.09 (1.01–1.18)	0.033 *	1.12 (1.03–1.21)	0.006 *	1.12 (1.04–1.21)	0.005 *

Abbreviations: CP, chronic periodontitis; RA, rheumatoid arthritis; SBP, systolic blood pressure; DBP, diastolic blood pressure; CCI, Charlson Comorbidity Index. * Conditional or unconditional logistic regression analysis, significance at *p* < 0.05. † Stratified model for age, sex, income, and region of residence. ‡ Model 1 was adjusted for smoking, alcohol consumption, obesity, and CCI scores. § Model 2 was adjusted for model 1 plus total cholesterol, systolic blood pressure, diastolic blood pressure, and fasting blood glucose.

## Data Availability

All data are available from the database of National Health Insurance Sharing Service (NHISS) https://nhiss.nhis.or.kr/ (accessed on 1 September 2023). NHISS allows access to all of this data at some processing charge for any researcher who promises to follow the research ethics guidelines. If you want to access the data used for this article, it may be downloaded from the aforementioned website after promising to follow the research ethics.

## Supplementary Materials

The following supporting information can be downloaded at: https://www.mdpi.com/article/10.3390/biomedicines12050936/s1. Table S1: Subgroup analyses of crude and adjusted odds ratios of chronic periodontitis (CP) for rheumatoid arthritis (RA) when participants are diagnosed with CP ≥ 1 within 1 year before the index date; Table S2: Subgroup analyses of crude and adjusted odds ratios of chronic periodontitis (CP) for rheumatoid arthritis (RA) when participants are diagnosed with CP ≥ 2 within 1 year before index date; Table S3: Subgroup analyses of crude and adjusted odds ratios of chronic periodontitis (CP) for rheumatoid arthritis (RA) when participants are diagnosed with CP ≥ 3 within 1 year before index date; Table S4: Subgroup analyses of crude and adjusted odds ratios of chronic periodontitis (CP) for rheumatoid arthritis (RA) when participants are diagnosed with CP ≥ 1 within 2 years before index date; Figure S1: Forest plots illustrating the adjusted odds ratio and corresponding 95% confidence intervals (CIs) for demographic, lifestyle, and comorbid factors related to chronic periodontitis (CP) concerning the development of rheumatoid arthritis (RA) when individuals are diagnosed with CP ≥ 1 within 1 year preceding the index date; Figure S2: Forest plots illustrating the adjusted odds ratio and corresponding 95% confidence intervals (CIs) for demographic, lifestyle, and comorbid factors related to chronic periodontitis (CP) concerning the development of rheumatoid arthritis (RA) when individuals are diagnosed with CP ≥ 2 within 1 year preceding the index date; Figure S3: Forest plots illustrating the adjusted odds ratio and corresponding 95% confidence intervals (CIs) for demographic, lifestyle, and comorbid factors related to chronic periodontitis (CP) concerning the development of rheumatoid arthritis (RA) when individuals are diagnosed with CP ≥ 3 within 1 year preceding the index date.
