# Supplementary material for: The Association of Chronic Periodontitis as a Potential Risk Factor with Rheumatoid Arthritis: A Nested Case-Control Study Using a Korean National Health Screening Cohort
Source: Biomedicines. 2024 Apr 23;12(5):936. doi: 10.3390/biomedicines12050936 (PMC11118670; doi:10.3390/biomedicines12050936)
Supplement: Supplementary file 1 [file biomedicines-12-00936-s001.zip › biomedicines-2950616-supplementary.pdf]

Supplementary Table S1. Subgroup analyses of crude and adjusted odds ratios of chronic periodontitis (CP) for rheumatoid arthritis (RA) when participants are diagnosed with CP ≥ 1 within 1 year before the index date.

	N of RA (exposure/total, %)	N of Control (exposure/total, %)	Odd ratios for RA (95% confidence interval)					
			Crude†	*p*	Model 1†‡	*p*	Model 2†§	*p*
Age < 65 years old (n = 8780)								
No CP	1424/1756 (81.1%)	5735/7024 (81.7%)	1		1		1	
CP ≥ 1	332/1756 (18.9%)	1289/7024 (18.4%)	1.04 (0.91–1.19)	0.589	1.04 (0.91–1.19)	0.578	1.04 (0.91–1.20)	0.534
Age ≥ 65 years old (n = 9060)								
No CP	1346/1812 (74.3%)	5484/7248 (75.7%)	1		1		1	
CP ≥ 1	466/1812 (25.7%)	1764/7248 (24.3%)	1.08 (0.96–1.21)	0.223	1.07 (0.95–1.21)	0.246	1.10 (0.97–1.24)	0.127
Men (n = 5275)								
No CP	770/1055 (73.0%)	3124/4220 (74.0%)	1		1		1	
CP ≥ 1	285/1055 (27.0%)	1096/4220 (26.0%)	1.06 (0.91–1.23)	0.491	1.06 (0.91–1.24)	0.434	1.08 (0.93–1.27)	0.307
Women (n = 12,565)								
No CP	2000/2513 (79.6%)	8095/10,052 (80.5%)	1		1		1	
CP ≥ 1	513/2513 (20.4%)	1957/10,052 (19.5%)	1.06 (0.95–1.18)	0.286	1.06 (0.95–1.18)	0.292	1.08 (0.96–1.20)	0.193
Low income (n = 8630)								
No CP	1370/1726 (79.4%)	5557/6904 (80.5%)	1		1		1	
CP ≥ 1	356/1726 (20.6%)	1347/6904 (19.5%)	1.07 (0.94–1.22)	0.298	1.08 (0.94–1.23)	0.269	1.09 (0.95–1.24)	0.209
High income (n = 9210)								
No CP	1400/1842 (76.0%)	5662/7368 (76.9%)	1		1		1	
CP ≥ 1	442/1842 (24.0%)	1706/7368 (23.2%)	1.05 (0.93–1.18)	0.445	1.05 (0.93–1.18)	0.473	1.06 (0.94–1.20)	0.312
Urban residents (n = 7705)								
No CP	1183/1541 (76.8%)	4726/6164 (76.7%)	1		1		1	
CP ≥ 1	358/1541 (23.2%)	1438/6164 (23.3%)	0.99 (0.87–1.14)	0.936	0.99 (0.87–1.13)	0.905	1.00 (0.87–1.14)	0.999
Rural residents (n = 10,135)								
No CP	1587/2027 (78.3%)	6493/8108 (80.1%)	1		1		1	
CP ≥ 1	440/2027 (21.7%)	1615/8108 (19.9%)	1.11 (0.99–1.26)	0.073	1.12 (0.99–1.26)	0.066	1.14 (1.01–1.29)	0.028
Underweight (n = 388)								
No CP	51/69 (73.9%)	257/319 (80.6%)	1		1		1	
CP ≥ 1	18/69 (26.1%)	62/319 (19.4%)	1.46 (0.80–2.68)	0.217	1.34 (0.72–2.51)	0.358	1.38 (0.73–2.61)	0.317
Normal weight (n = 6468)								
No CP	1045/1334 (78.3%)	4076/5134 (79.4%)	1		1		1	
CP ≥ 1	289/1334 (21.7%)	1058/5134 (20.6%)	1.07 (0.92–1.23)	0.397	1.06 (0.92–1.23)	0.418	1.09 (0.94–1.26)	0.271
Overweight (n = 4807)								
No CP	721/945 (76.3%)	3019/3862 (78.2%)	1		1		1	
CP ≥ 1	224/945 (23.7%)	843/3862 (21.8%)	1.11 (0.94–1.32)	0.214	1.11 (0.94–1.32)	0.223	1.13 (0.95–1.34)	0.172
Obese (n = 6177)								
No CP	953/1220 (78.1%)	3867/4957 (78.0%)	1		1		1	
CP ≥ 1	267/1220 (21.9%)	1090/4957 (22.0%)	0.99 (0.85–1.16)	0.938	1.00 (0.86–1.17)	1.000	1.01 (0.86–1.17)	0.782
Non-smoker (n = 14,404)								
No CP	2241/2853 (78.6%)	9183/11,551 (79.5%)	1		1		1	
CP ≥ 1	612/2853 (21.5%)	2368/11,551 (20.5%)	1.06 (0.96–1.17)	0.262	1.06 (0.96–1.17)	0.244	1.08 (0.97–1.19)	0.159
Past smoker and current smoker (n = 3436)								
No CP	529/715 (74.0%)	2036/2721 (74.8%)	1		1		1	
CP ≥ 1	186/715 (26.0%)	685/2721 (25.2%)	1.05 (0.87–1.26)	0.644	1.05 (0.87–1.27)	0.602	1.08 (0.89–1.30)	0.455
Alcohol consumption < 1 time a week (n = 13,182)								
No CP	2142/2690 (79.6%)	8459/10,492 (80.6%)	1		1		1	
CP ≥ 1	548/2690 (20.4%)	2033/10,492 (19.4%)	1.06 (0.96–1.18)	0.246	1.06 (0.96–1.18)	0.266	1.08 (0.97–1.20)	0.182
Alcohol consumption ≥ 1 time a week (n = 4658)								
No CP	628/878 (71.5%)	2760/3780 (73.0%)	1		1		1	
CP ≥ 1	250/878 (28.5%)	1020/3780 (27.0%)	1.08 (0.91–1.27)	0.372	1.08 (0.91–1.27)	0.374	1.08 (0.91–1.27)	0.376
SBP < 140 mmHg and DBP < 90 mmHg (n = 13,526)								
No CP	2143/2783 (77.0%)	8396/10,743 (78.2%)	1		1		1	
CP ≥ 1	640/2783 (23.0%)	2347/10,743 (21.9%)	1.07 (0.97–1.18)	0.193	1.07 (0.97–1.18)	0.178	1.09 (0.99–1.21)	0.086
SBP ≥ 140 mmHg or DBP ≥ 90 mmHg (n = 4314)								
No CP	627/785 (79.9%)	2823/3529 (80.0%)	1		1		1	
CP ≥ 1	158/785 (20.1%)	706/3529 (20.0%)	1.01 (0.83–1.22)	0.939	1.01 (0.83–1.23)	0.925	1.02 (0.84–1.24)	0.871
Fasting blood glucose < 100 mg/dL (n = 11,535)								
No CP	627/785 (79.9%)	2,823/3,529 (80.0%)	1		1		1	
CP ≥ 1	158/785 (20.1%)	706/3,529 (20.0%)	1.11 (0.99–1.23)	0.074	1.10 (0.99–1.23)	0.088	1.12 (1.00–1.25)	0.055
Fasting blood glucose ≥ 100 mg/dL (n = 6305)								

No CP	906/1187 (76.3%)	3901/5118 (76.2%)	1		1		1	
CP ≥ 1	281/1187 (23.7%)	1217/5118 (23.8%)	0.99 (0.86–1.15)	0.939	0.99 (0.85–1.15)	0.921	1.01 (0.87–1.18)	0.870
Total cholesterol < 200mg/dL (n = 9209)								
No CP	1479/1907 (77.6%)	5717/7302 (78.3%)	1		1		1	
CP ≥ 1	428/1907 (22.4%)	1585/7302 (21.7%)	1.04 (0.93–1.18)	0.485	1.05 (0.93–1.18)	0.454	1.06 (0.94–1.20)	0.369
Total cholesterol ≥ 200mg/dL (n = 8631)								
No CP	1291/1661 (77.7%)	5502/6970 (78.9%)	1		1		1	
CP ≥ 1	370/1661 (22.3%)	1468/6970 (21.1%)	1.07 (0.94–1.22)	0.278	1.08 (0.94–1.22)	0.273	1.10 (0.96–1.25)	0.156
CCI scores = 0 (n = 11,036)								
No CP	1525/1991 (76.6%)	7105/9045 (78.6%)	1		1		1	
CP ≥ 1	466/1991 (23.4%)	1940/9045 (21.5%)	1.12 (1.00–1.26)	0.056	1.13 (1.00–1.27)	0.043*	1.13 (1.01–1.27)	0.038*
CCI score = 1 (n = 3038)								
No CP	588/740 (79.5%)	1782/2298 (77.6%)	1		1		1	
CP ≥ 1	152/740 (20.5%)	516/2298 (22.5%)	0.89 (0.73–1.09)	0.275	0.90 (0.74–1.11)	0.328	0.91 (0.74–1.11)	0.345
CCI score ≥ 2 (n = 3766)								
No CP	657/837 (78.5%)	2332/2929 (79.6%)	1		1		1	
CP ≥ 1	180/837 (21.5%)	597/2929 (20.4%)	1.07 (0.89–1.29)	0.479	1.08 (0.90–1.31)	0.399	1.10 (0.91–1.33)	0.326

Abbreviations: CP, chronic periodontitis; RA, rheumatoid arthritis; SBP, systolic blood pressure; DBP, diastolic blood pressure; CCI, Charlson Comorbidity Index.

* Conditional or unconditional logistic regression analysis, Significance at $p < 0.05$

† Stratified model for age, sex, income, and region of residence.

‡ Model 1 was adjusted for smoking, alcohol consumption, obesity and CCI scores.

§ Model 2 was adjusted for model 1 plus total cholesterol, systolic blood pressure, diastolic blood pressure, and fasting blood glucose.

Supplementary Table S2. Subgroup analyses of crude and adjusted odds ratios of chronic periodontitis (CP) for rheumatoid arthritis (RA) when participants are diagnosed with CP ≥ 2 within 1 year before index date.

	N of RA (exposure/total, %)	N of Control (exposure/total, %)	Odd ratios for RA (95% confidence interval)					
			**Crude†**	***p***	**Model 1†‡**	***p***	**Model 2†§**	***p***
Age < 65 years old (n = 8780)								
CP < 2	1604/1756 (91.3%)	6423/7024 (91.4%)	1		1		1	
CP ≥ 2	152/1756 (8.7%)	601/7024 (8.6%)	1.01 (0.84–1.22)	0.894	1.01 (0.84–1.22)	0.896	1.01 (0.84–1.22)	0.904
Age ≥ 65 years old (n = 9060)								
CP < 2	1574/1812 (86.9%)	6344/7248 (87.5%)	1		1		1	
CP ≥ 2	238/1812 (13.1%)	904/7248 (12.5%)	1.06 (0.91–1.24)	0.448	1.06 (0.90–1.23)	0.494	1.08 (0.92–1.26)	0.340
Men (n = 5275)								
CP < 2	898/1055 (85.1%)	3649/4220 (86.5%)	1		1		1	
CP ≥ 2	157/1055 (14.9%)	571/4220 (13.5%)	1.12 (0.92–1.35)	0.255	1.12 (0.93–1.36)	0.244	1.14 (0.94–1.38)	0.188
Women (n = 12,565)								
CP < 2	2280/2513 (90.7%)	9118/10,052 (90.7%)	1		1		1	
CP ≥ 2	233/2513 (9.3%)	934/10,052 (9.3%)	1.00 (0.86–1.16)	0.976	0.99 (0.86–1.16)	0.945	1.01 (0.86–1.17)	0.942
Low income (n = 8630)								
CP < 2	1554/1726 (90.0%)	6256/6904 (90.6%)	1		1		1	
CP ≥ 2	172/1726 (10.0%)	648/6904 (9.4%)	1.07 (0.90–1.28)	0.463	1.07 (0.89–1.28)	0.469	1.07 (0.90–1.28)	0.431
High income (n = 9210)								
CP < 2	1624/1842 (88.2%)	6511/7368 (88.4%)	1		1		1	
CP ≥ 2	218/1842 (11.8%)	857/7368 (11.6%)	1.02 (0.87–1.19)	0.807	1.02 (0.87–1.19)	0.829	1.04 (0.89–1.22)	0.642
Urban residents (n = 7705)								
CP < 2	1365/1541 (88.6%)	5434/6164 (88.2%)	1		1		1	
CP ≥ 2	176/1541 (11.4%)	730/6164 (11.8%)	0.96 (0.81–1.14)	0.649	0.96 (0.80–1.14)	0.625	0.96 (0.80–1.15)	0.648
Rural residents (n = 10,135)								
CP < 2	1813/2027 (89.4%)	7333/8108 (90.4%)	1		1		1	
CP ≥ 2	214/2027 (10.6%)	775/8108 (9.6%)	1.12 (0.95–1.31)	0.175	1.12 (0.95–1.31)	0.176	1.14 (0.97–1.34)	0.107
Underweight (n = 388)								
CP < 2	62/69 (89.9%)	288/319 (90.3%)	1		1		1	
CP ≥ 2	7/69 (10.1%)	31/319 (9.7%)	1.05 (0.44–2.49)	0.914	0.88 (0.36–2.13)	0.769	0.86 (0.35–2.13)	0.741
Normal weight (n = 6468)								
CP < 2	1179/1334 (88.4%)	4610/5134 (89.8%)	1		1		1	
CP ≥ 2	155/1334 (11.6%)	524/5134 (10.2%)	1.16 (0.96–1.40)	0.134	1.15 (0.95–1.40)	0.144	1.17 (0.97–1.42)	0.110
Overweight (n = 4807)								
CP < 2	858/945 (90.8%)	3451/3862 (89.4%)	1		1		1	
CP ≥ 2	87/945 (9.2%)	411/3862 (10.6%)	0.85 (0.67–1.09)	0.195	0.84 (0.66–1.07)	0.166	0.85 (0.66–1.08)	0.185
Obese (n = 6177)								
CP < 2	1079/1220 (88.4%)	4418/4957 (89.1%)	1		1		1	
CP ≥ 2	141/1220 (11.6%)	539/4957 (10.9%)	1.07 (0.88–1.30)	0.494	1.08 (0.88–1.31)	0.473	1.09 (0.89–1.33)	0.400
Non-smoker (n = 14,404)								
CP < 2	2570/2853 (90.1%)	10,418/11,551 (90.2%)	1		1		1	
CP ≥ 2	283/2853 (9.9%)	1133/11,551 (9.8%)	1.01 (0.88–1.16)	0.858	1.01 (0.88–1.16)	0.858	1.03 (0.89–1.18)	0.702
Past smoker and current smoker (n = 3436)								
CP < 2	608/715 (85.0%)	2349/2721 (86.3%)	1		1		1	
CP ≥ 2	107/715 (15.0%)	372/2721 (13.7%)	1.11 (0.88–1.40)	0.374	1.12 (0.89–1.42)	0.338	1.14 (0.90–1.45)	0.273
Alcohol consumption < 1 time a week (n = 13,182)								
CP < 2	2435/2690 (90.5%)	9483/10,492 (90.4%)	1		1		1	
CP ≥ 2	255/2690 (9.5%)	1009/10,492 (9.6%)	0.98 (0.85–1.14)	0.830	0.98 (0.84–1.13)	0.752	0.99 (0.85–1.14)	0.865
Alcohol consumption ≥ 1 time a week (n = 4658)								
CP < 2	743/878 (84.6%)	3284/3780 (86.9%)	1		1		1	
CP ≥ 2	135/878 (15.4%)	496/3780 (13.1%)	1.20 (0.98–1.48)	0.079	1.20 (0.98–1.48)	0.079	1.20 (0.98–1.48)	0.081
SBP < 140 mmHg and DBP < 90 mmHg (n = 13,526)								
CP < 2	2472/2783 (88.8%)	9583/10,743 (89.2%)	1		1		1	
CP ≥ 2	311/2783 (11.2%)	1160/10,743 (10.8%)	1.04 (0.91–1.19)	0.565	1.03 (0.91–1.18)	0.615	1.05 (0.92–1.20)	0.467
SBP ≥ 140 mmHg or DBP ≥ 90 mmHg (n = 4314)								
CP < 2	706/785 (89.9%)	3184/3529 (90.2%)	1		1		1	
CP ≥ 2	79/785 (10.1%)	345/3529 (9.8%)	1.03 (0.80–1.34)	0.805	1.04 (0.80–1.35)	0.760	1.04 (0.80–1.36)	0.747
Fasting blood glucose < 100 mg/dL (n = 11,535)								
CP < 2	2134/2381 (89.6%)	8242/9154 (90.0%)	1		1		1	
CP ≥ 2	247/2381 (10.4%)	912/9154 (10.0%)	1.05 (0.90–1.21)	0.548	1.04 (0.89–1.21)	0.627	1.05 (0.90–1.22)	0.548
Fasting blood glucose ≥ 100 mg/dL (n = 6305)								

CP < 2	1044/1187 (88.0%)	4525/5118 (88.4%)	1		1		1	
CP ≥ 2	143/1187 (12.1%)	593/5118 (11.6%)	1.05 (0.86–1.27)	0.653	1.04 (0.86–1.27)	0.673	1.07 (0.87–1.30)	0.528
Total cholesterol < 200mg/dL (n = 9209)								
CP < 2	1691/1907 (88.7%)	6524/7302 (89.4%)	1		1		1	
CP ≥ 2	216/1907 (11.3%)	778/7302 (10.7%)	1.07 (0.91–1.26)	0.400	1.07 (0.91–1.26)	0.390	1.08 (0.92–1.27)	0.353
Total cholesterol ≥ 200mg/dL (n = 8631)								
CP < 2	1487/1661 (89.5%)	6243/6970 (89.6%)	1		1		1	
CP ≥ 2	174/1661 (10.5%)	727/6970 (10.4%)	1.00 (0.84–1.20)	0.957	1.00 (0.84–1.20)	0.968	1.03 (0.86–1.22)	0.774
CCI score = 0 (n = 11,036)								
CP < 2	1757/1991 (88.3%)	8089/9045 (89.4%)	1		1		1	
CP ≥ 2	234/1991 (11.8%)	956/9045 (10.6%)	1.13 (0.97–1.31)	0.123	1.14 (0.97–1.32)	0.103	1.14 (0.97–1.32)	0.106
CCI score = 1 (n = 3038)								
CP < 2	669/740 (90.4%)	2047/2298 (89.1%)	1		1		1	
CP ≥ 2	71/740 (9.6%)	251/2298 (10.9%)	0.87 (0.66–1.14)	0.308	0.87 (0.66–1.15)	0.336	0.87 (0.66–1.15)	0.336
CCI score ≥ 2 (n = 3766)								
CP < 2	752/837 (89.8%)	2631/2929 (89.8%)	1		1		1	
CP ≥ 2	85/837 (10.2%)	298/2929 (10.2%)	1.00 (0.77–1.29)	0.987	1.00 (0.77–1.29)	0.997	1.00 (0.77–1.29)	0.987

Abbreviations: CP, chronic periodontitis; RA, rheumatoid arthritis; SBP, systolic blood pressure; DBP, diastolic blood pressure; CCI, Charlson Comorbidity Index.

* Conditional or unconditional logistic regression analysis, Significance at $p < 0.05$

† Stratified model for age, sex, income, and region of residence.

‡ Model 1 was adjusted for smoking, alcohol consumption, obesity and CCI scores.

§ Model 2 was adjusted for model 1 plus total cholesterol, systolic blood pressure, diastolic blood pressure, and fasting blood glucose.

Supplementary Table S3. Subgroup analyses of crude and adjusted odds ratios of chronic periodontitis (CP) for rheumatoid arthritis (RA) when participants are diagnosed with CP ≥ 3 within 1 year before index date.

	N of RA (exposure/total, %)	N of Control (exposure/total, %)	Odd ratios for RA (95% confidence interval)				
			Crude†	*p*	Model 1†‡	*p*	Model 2†§	*p*
Age < 65 years old (n = 8780)								
CP < 3	1675/1756 (95.4%)	6669/7024 (95.0%)	1		1		1	
CP ≥ 3	81/1756 (4.6%)	355/7024 (5.1%)	0.91 (0.71–1.16)	0.447	0.91 (0.71–1.17)	0.458	0.91 (0.71–1.17)	0.455
Age ≥ 65 years old (n = 9060)								
CP < 3	1673/1812 (92.3%)	6732/7248 (92.9%)	1		1		1	
CP ≥ 3	139/1812 (7.7%)	516/7248 (7.1%)	1.08 (0.89–1.32)	0.417	1.08 (0.88–1.31)	0.468	1.10 (0.91–1.34)	0.331
Men (n = 5275)								
CP < 3	963/1055 (91.3%)	3885/4220 (92.1%)	1		1		1	
CP ≥ 3	92/1055 (8.7%)	335/4220 (7.9%)	1.11 (0.87–1.41)	0.405	1.12 (0.88–1.42)	0.370	1.14 (0.89–1.45)	0.299
Women (n = 12,565)								
CP < 3	2385/2513 (94.9%)	9516/10,052 (94.7%)	1		1		1	
CP ≥ 3	128/2513 (5.1%)	536/10,052 (5.3%)	0.95 (0.78–1.16)	0.637	0.95 (0.78–1.16)	0.618	0.96 (0.79–1.17)	0.709
Low income (n = ,630)								
CP < 3	1630/1726 (94.4%)	6529/6904 (94.6%)	1		1		1	
CP ≥ 3	96/1726 (5.6%)	375/6904 (5.4%)	1.03 (0.81–1.29)	0.830	1.03 (0.81–1.29)	0.834	1.04 (0.82–1.31)	0.760
High income (n = 9210)								
CP < 3	1718/1842 (93.3%)	6872/7368 (93.3%)	1		1		1	
CP ≥ 3	124/1842 (6.7%)	496/7368 (6.7%)	1.00 (0.82–1.23)	1.000	1.00 (0.81–1.22)	0.981	1.02 (0.83–1.25)	0.839
Urban residents (n = 7705)								
CP < 3	1434/1541 (93.1%)	5,737/6164 (93.1%)	1		1		1	
CP ≥ 3	107/1541 (6.9%)	427/6164 (6.9%)	1.00 (0.80–1.25)	0.982	1.00 (0.80–1.25)	0.992	1.00 (0.80–1.25)	0.996
Rural residents (n = 10,135)								
CP < 3	1914/2027 (94.4%)	7,664/8108 (94.5%)	1		1		1	
CP ≥ 3	113/2027 (5.6%)	444/8108 (5.5%)	1.02 (0.82–1.26)	0.861	1.02 (0.82–1.26)	0.849	1.05 (0.85–1.30)	0.651
Underweight (n = 388)								
CP < 3	64/69 (92.8%)	305/319 (95.6%)	1		1		1	
CP ≥ 3	5/69 (7.3%)	14/319 (4.4%)	1.70 (0.59–4.89)	0.324	1.55 (0.52–4.58)	0.432	1.58 (0.53–4.73)	0.416
Normal weight (n = 6468)								
CP < 3	1247/1334 (93.5%)	4,840/5134 (94.3%)	1		1		1	
CP ≥ 3	87/1334 (6.5%)	294/5134 (5.7%)	1.15 (0.90–1.47)	0.272	1.14 (0.89–1.46)	0.303	1.16 (0.91–1.49)	0.239
Overweight (n = 4807)								
CP < 3	898/945 (95.0%)	3606/3862 (93.4%)	1		1		1	
CP ≥ 3	47/945 (5.0%)	256/3862 (6.6%)	0.74 (0.54–1.01)	0.062	0.73 (0.53–1.01)	0.057	0.74 (0.54–1.02)	0.066
Obese (n = 6177)								
CP < 3	1139/1220 (93.4%)	4650/4957 (93.8%)	1		1		1	
CP ≥ 3	81/1220 (6.6%)	307/4957 (6.2%)	1.08 (0.84–1.39)	0.565	1.09 (0.85–1.41)	0.504	1.10 (0.85–1.43)	0.450
Non-smoker (n = 14,404)								
CP < 3	2693/2853 (94.4%)	10,904/11,551 (94.4%)	1		1		1	
CP ≥ 3	160/2853 (5.6%)	647/11,551 (5.6%)	1.00 (0.84–1.20)	0.989	1.00 (0.84–1.20)	0.999	1.02 (0.85–1.22)	0.854
Past smoker and current smoker (n = 3436)								
CP < 3	655/715 (91.6%)	2497/2721 (91.8%)	1		1		1	
CP ≥ 3	60/715 (8.4%)	224/2721 (8.2%)	1.02 (0.76–1.38)	0.890	1.04 (0.77–1.40)	0.819	1.06 (0.78–1.43)	0.724
Alcohol consumption < 1 time a week (n = 13,182)								
CP < 3	2548/2690 (94.7%)	9926/10,492 (94.6%)	1		1		1	
CP ≥ 3	142/2690 (5.3%)	566/10,492 (5.4%)	0.98 (0.81–1.18)	0.813	0.97 (0.80–1.17)	0.747	0.98 (0.81–1.19)	0.864
Alcohol consumption ≥ 1 time a week (n = 4658)								
CP < 3	800/878 (91.1%)	3475/3780 (91.9%)	1		1		1	
CP ≥ 3	78/878 (8.9%)	305/3780 (8.1%)	1.11 (0.86–1.44)	0.429	1.11 (0.86–1.45)	0.419	1.11 (0.86–1.45)	0.426
SBP < 140 mmHg and DBP < 90 mmHg (n = 13,526)								
CP < 3	2603/2783 (93.5%)	10,068/10,743 (93.7%)	1		1		1	
CP ≥ 3	180/2783 (6.5%)	675/10,743 (6.3%)	1.03 (0.87–1.22)	0.719	1.03 (0.87–1.22)	0.744	1.05 (0.88–1.24)	0.592
SBP ≥ 140 mmHg or DBP ≥ 90 mmHg (n = 4314)								
CP < 3	745/785 (94.9%)	3333/3529 (94.5%)	1		1		1	
CP ≥ 3	40/785 (5.1%)	196/3529 (5.6%)	0.91 (0.64–1.29)	0.610	0.92 (0.65–1.30)	0.624	0.92 (0.64–1.30)	0.626
Fasting blood glucose < 100 mg/dL (n = 11,535)								
CP < 3	2240/2381 (94.1%)	8636/9154 (94.3%)	1		1		1	
CP ≥ 3	141/2381 (5.9%)	518/9154 (5.7%)	1.05 (0.87–1.27)	0.618	1.04 (0.86–1.26)	0.678	1.05 (0.87–1.28)	0.593
Fasting blood glucose ≥ 100 mg/dL (n = 6305)								

CP < 3	1108/1187 (93.3%)	4765/5118 (93.1%)	1		1		1	
CP ≥ 3	79/1187 (6.7%)	353/5118 (6.9%)	0.96 (0.75–1.24)	0.769	0.96 (0.75–1.24)	0.757	0.99 (0.76–1.27)	0.908
Total cholesterol < 200mg/dL (n = 9209)								
CP < 3	1778/1907 (93.2%)	6856/7302 (93.9%)	1		1		1	
CP ≥ 3	129/1907 (6.8%)	446/7302 (6.1%)	1.12 (0.91–1.37)	0.292	1.12 (0.91–1.37)	0.274	1.13 (0.92–1.38)	0.246
Total cholesterol ≥ 200mg/dL (n = 8631)								
CP < 3	1570/1661 (94.5%)	6545/6970 (93.9%)	1		1		1	
CP ≥ 3	91/1661 (5.5%)	425/6970 (6.1%)	0.89 (0.71–1.13)	0.339	0.89 (0.70–1.12)	0.315	0.91 (0.72–1.15)	0.447
CCI score = 0 (n = 11,036)								
CP < 3	1859/1991 (93.4%)	8481/9045 (93.8%)	1		1		1	
CP ≥ 3	132/1991 (6.6%)	564/9045 (6.2%)	1.07 (0.88–1.30)	0.512	1.08 (0.88–1.31)	0.462	1.08 (0.88–1.31)	0.468
CCI score = 1 (n = 3038)								
CP < 3	699/740 (94.5%)	2162/2298 (94.1%)	1		1		1	
CP ≥ 3	41/740 (5.5%)	136/2298 (5.9%)	0.93 (0.65–1.34)	0.703	0.94 (0.66–1.35)	0.747	0.95 (0.66–1.36)	0.766
CCI score ≥ 2 (n = 3766)								
CP < 3	790/837 (94.4%)	2758/2929 (94.2%)	1		1		1	
CP ≥ 3	47/837 (5.6%)	171/2929 (5.8%)	0.96 (0.69–1.34)	0.810	0.96 (0.68–1.34)	0.791	0.96 (0.69–1.34)	0.810

Abbreviations: CP, chronic periodontitis; RA, rheumatoid arthritis; SBP, systolic blood pressure; DBP, diastolic blood pressure; CCI, Charlson Comorbidity Index.

* Conditional or unconditional logistic regression analysis, Significance at $p < 0.05$

† Stratified model for age, sex, income, and region of residence.

‡ Model 1 was adjusted for smoking, alcohol consumption, obesity and CCI scores.

§ Model 2 was adjusted for model 1 plus total cholesterol, systolic blood pressure, diastolic blood pressure, and fasting blood glucose.

Supplementary Table S4. Subgroup analyses of crude and adjusted odds ratios of chronic periodontitis (CP) for rheumatoid arthritis (RA) when participants are diagnosed with CP ≥ 1 within 2 years before index date.

	N of RA (exposure/total, %)	N of Control (exposure/total, %)	Odd ratios for RA (95% confidence interval)					
			**Crude†**	*p*	**Model 1†‡**	*p*	**Model 2†§**	*p*
Age < 65 years old (n = 8780)								
No CP	1,229/1756 (70.0%)	4961/7024 (70.6%)	1		1		1	
CP ≥ 1	527/1756 (30.0%)	2063/7024 (29.4%)	1.03 (0.92–1.16)	0.597	1.03 (0.92–1.16)	0.596	1.04 (0.93–1.17)	0.509
Age ≥ 65 years old (n = 9060)								
No CP	1087/1812 (60.0%)	4568/7248 (63.0%)	1		1		1	
CP ≥ 1	725/1812 (40.0%)	2680/7248 (37.0%)	1.14 (1.02–1.26)	0.017*	1.14 (1.02–1.26)	0.018*	1.17 (1.05–1.30)	0.005*
Men (n = 5275)								
No CP	617/1055 (58.5%)	2555/4220 (60.6%)	1		1		1	
CP ≥ 1	438/1055 (41.5%)	1665/4220 (39.5%)	1.09 (0.95–1.25)	0.221	1.09 (0.95–1.25)	0.217	1.11 (0.97–1.28)	0.127
Women (n = 12,565)								
No CP	1699/2513 (67.6%)	6974/10,052 (69.4%)	1		1		1	
CP ≥ 1	814/2513 (32.4%)	3078/10,052 (30.6%)	1.09 (0.99–1.19)	0.086	1.09 (0.99–1.20)	0.081	1.11 (1.01–1.22)	0.037*
Low income (n = 8630)								
No CP	1159/1726 (67.2%)	4756/6904 (68.9%)	1		1		1	
CP ≥ 1	567/1726 (32.9%)	2148/6904 (31.1%)	1.08 (0.97–1.21)	0.164	1.09 (0.97–1.22)	0.144	1.10 (0.98–1.24)	0.092
High income (n = 9210)								
No CP	1157/1842 (62.8%)	4773/7368 (64.8%)	1		1		1	
CP ≥ 1	685/1842 (37.2%)	2595/7368 (35.2%)	1.09 (0.98–1.21)	0.115	1.09 (0.98–1.21)	0.120	1.11 (1.00–1.24)	0.053
Urban residents (n = 7705)								
No CP	971/1541 (63.0%)	3971/6164 (64.4%)	1		1		1	
CP ≥ 1	570/1541 (37.0%)	2193/6164 (35.6%)	1.06 (0.95–1.19)	0.302	1.06 (0.94–1.19)	0.329	1.07 (0.95–1.21)	0.243
Rural residents (n = 10,135)								
No CP	1345/2027 (66.4%)	5558/8108 (68.6%)	1		1		1	
CP ≥ 1	682/2027 (33.7%)	2550/8108 (31.5%)	1.11 (1.00–1.23)	0.058	1.11 (1.00–1.23)	0.048*	1.14 (1.02–1.26)	0.018*
Underweight (n = 388)								
No CP	48/69 (69.6%)	224/319 (70.2%)	1		1		1	
CP ≥ 1	21/69 (30.4%)	95/319 (29.8%)	1.03 (0.59–1.82)	0.914	0.97 (0.54–1.75)	0.931	1.03 (0.59–1.82)	0.995
Normal weight (n = 6468)								
No CP	872/1334 (65.4%)	3477/5134 (67.7%)	1		1		1	
CP ≥ 1	462/1334 (34.6%)	1657/5134 (32.3%)	1.11 (0.98–1.26)	0.102	1.11 (0.98–1.26)	0.111	1.14 (1.10–1.30)	0.049*
Overweight (n = 4807)								
No CP	602/945 (63.7%)	2557/3862 (66.2%)	1		1		1	
CP ≥ 1	343/945 (36.3%)	1305/3862 (33.8%)	1.12 (0.96–1.30)	0.146	1.11 (0.96–1.29)	0.164	1.13 (0.97–1.31)	0.116
Obese (n = 6177)								
No CP	794/1220 (65.1%)	3271/4957 (66.0%)	1		1		1	
CP ≥ 1	426/1220 (34.9%)	1686/4957 (34.0%)	1.04 (0.91–1.19)	0.549	1.05 (0.92–1.20)	0.449	1.06 (0.93–1.22)	0.374
Non-smoker (n = 14,404)								
No CP	1885/2853 (66.1%)	7847/11,551 (67.9%)	1		1		1	
CP ≥ 1	968/2853 (33.9%)	3704/11,551 (32.1%)	1.09 (1.00–1.19)	0.057	1.09 (1.00–1.19)	0.048*	1.11 (1.01–1.21)	0.023*
Past smoker and current smoker (n = 3436)								
No CP	431/715 (60.3%)	1682/2721 (61.8%)	1		1		1	
CP ≥ 1	284/715 (39.7%)	1039/2721 (38.2%)	1.07 (0.90–1.26)	0.453	1.08 (0.91–1.28)	0.400	1.11 (0.93–1.31)	0.254
Alcohol consumption < 1 time a week (n = 13,182)								
No CP	1825/2690 (67.8%)	7314/10,492 (69.7%)	1		1		1	
CP ≥ 1	865/2690 (32.2%)	3178/10,492 (30.3%)	1.09 (1.00–1.19)	0.061	1.09 (0.99–1.19)	0.066	1.10 (1.01–1.21)	0.037*
Alcohol consumption ≥ 1 time a week (n = 4658)								
No CP	491/878 (55.9%)	2215/3780 (58.6%)	1		1		1	
CP ≥ 1	387/878 (44.1%)	1565/3780 (41.4%)	1.12 (0.96–1.29)	0.148	1.11 (0.96–1.29)	0.159	1.12 (0.96–1.30)	0.148
SBP < 140 mmHg and DBP < 90 mmHg (n = 13,526)								
No CP	1778/2783 (63.9%)	7101/10,743 (66.1%)	1		1		1	
CP ≥ 1	1005/2783 (36.1%)	3642/10,743 (33.9%)	1.10 (1.01–1.20)	0.029*	1.11 (1.01–1.21)	0.025*	1.13 (1.04–1.24)	0.007*
SBP ≥ 140 mmHg or DBP ≥ 90 mmHg (n = 4314)								
No CP	538/785 (68.5%)	2428/3529 (68.8%)	1		1		1	
CP ≥ 1	247/785 (31.5%)	1101/3529 (31.2%)	1.01 (0.86–1.20)	0.884	1.02 (0.86–1.20)	0.853	1.02 (0.86–1.21)	0.782
Fasting blood glucose < 100 mg/dL (n = 11,535)								
No CP	1561/2381 (65.6%)	6272/9154 (68.5%)	1		1		1	
CP ≥ 1	820/2381 (34.4%)	2882/9154 (31.5%)	1.14 (1.04–1.26)	0.006*	1.14 (1.03–1.25)	0.008*	1.15 (1.05–1.27)	0.004*
Fasting blood glucose ≥ 100 mg/dL (n = 6305)								

No CP	755/1187 (63.6%)	3257/5118 (63.6%)	1		1		1	
CP ≥ 1	432/1187 (36.4%)	1861/5118 (36.4%)	1.00 (0.88–1.14)	0.983	1.00 (0.88–1.14)	0.975	1.03 (0.90–1.18)	0.677
Total cholesterol < 200mg/dL (n = 9209)								
No CP	1245/1907 (65.3%)	4853/7302 (66.5%)	1		1		1	
CP ≥ 1	662/1907 (34.7%)	2449/7302 (33.5%)	1.05 (0.95–1.17)	0.334	1.06 (0.95–1.18)	0.311	1.07 (0.96–1.19)	0.217
Total cholesterol ≥ 200mg/dL (n = 8631)								
No CP	1071/1661 (64.5%)	4676/6970 (67.1%)	1		1		1	
CP ≥ 1	590/1661 (35.5%)	2294/6970 (32.9%)	1.12 (1.00–1.26)	0.043*	1.13 (1.01–1.26)	0.040*	1.15 (1.03–1.29)	0.015*
CCI score = 0 (n = 11,036)								
No CP	1266/1991 (63.6%)	6023/9045 (66.6%)	1		1		1	
CP ≥ 1	725/1991 (36.4%)	3022/9045 (33.4%)	1.14 (1.03–1.26)	0.010*	1.15 (1.04–1.28)	0.007*	1.16 (1.05–1.29)	0.005*
CCI score = 1 (n = 3038)								
No CP	487/740 (65.8%)	1513/2298 (65.8%)	1		1		1	
CP ≥ 1	253/740 (34.2%)	785/2298 (34.2%)	1.00 (0.84–1.19)	0.988	1.02 (0.85–1.21)	0.863	1.02 (0.86–1.22)	0.806
CCI score ≥ 2 (n = 3766)								
No CP	563/837 (67.3%)	1993/2929 (68.0%)	1		1		1	
CP ≥ 1	274/837 (32.7%)	936/2929 (32.0%)	1.04 (0.88–1.22)	0.669	1.04 (0.89–1.23)	0.604	1.06 (0.90–1.25)	0.495

Abbreviations: CP, chronic periodontitis; RA, rheumatoid arthritis; SBP, systolic blood pressure; DBP, diastolic blood pressure; CCI, Charlson Comorbidity Index.

* Conditional or unconditional logistic regression analysis, Significance at $p < 0.05$

† Stratified model for age, sex, income, and region of residence.

‡ Model 1 was adjusted for smoking, alcohol consumption, obesity and CCI scores.

§ Model 2 was adjusted for model 1 plus total cholesterol, systolic blood pressure, diastolic blood pressure, and fasting blood glucose.

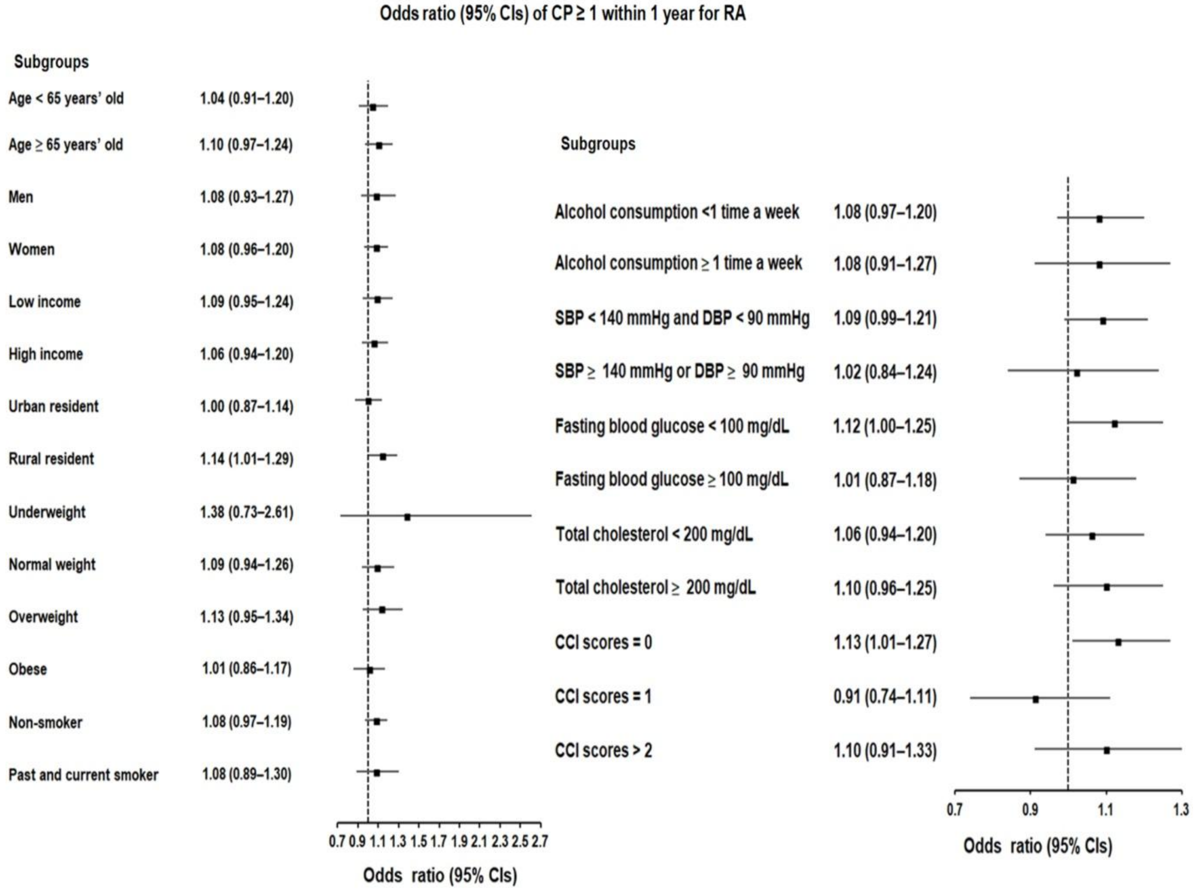

Supplementary Figure S1. Forest plots illustrating the adjusted odds ratio and corresponding 95% confidence intervals (CIs) for demographic, lifestyle, and comorbid factors related to chronic periodontitis (CP) concerning the development of rheumatoid arthritis (RA) when individuals are diagnosed with CP ≥ 1 within 1 year preceding the index date.

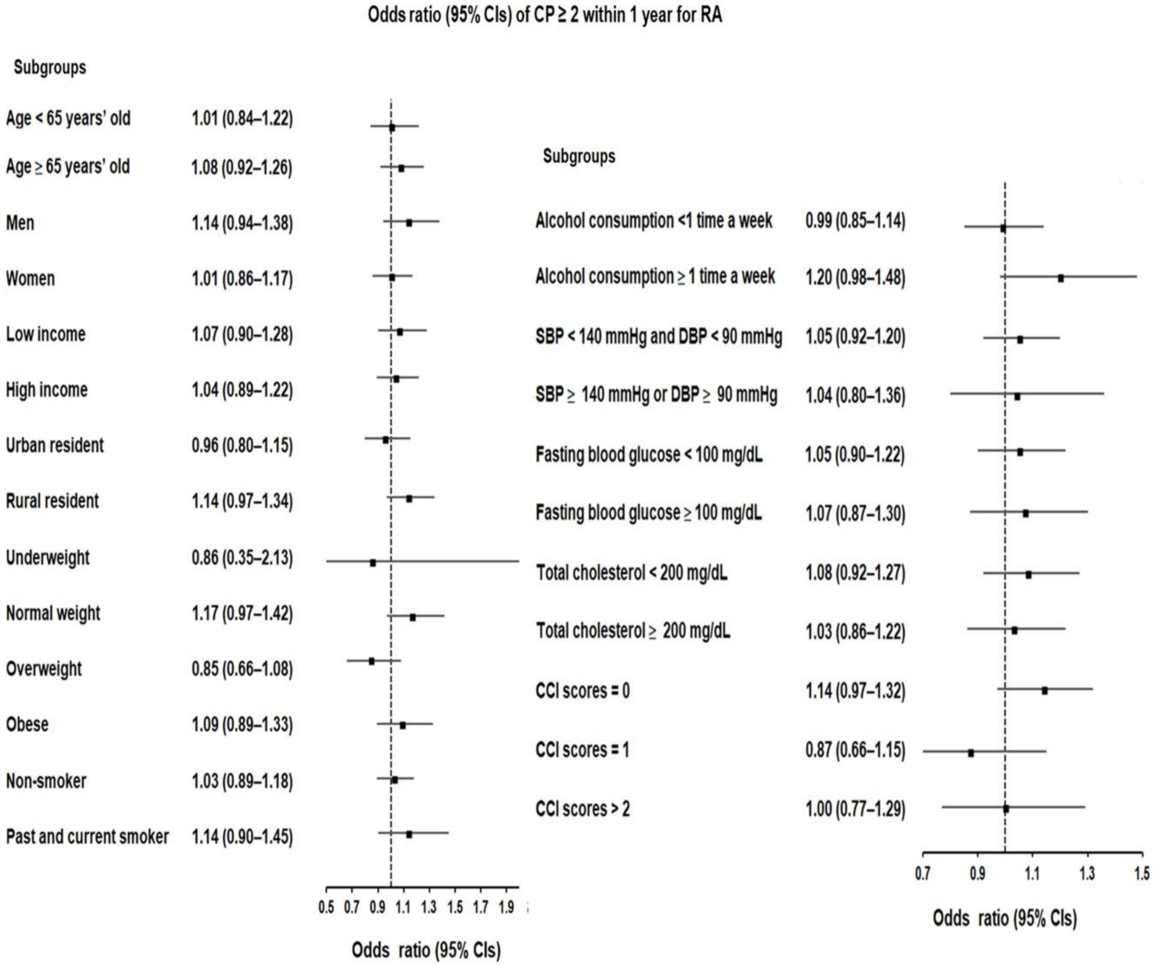

Supplementary Figure S2. Forest plots illustrating the adjusted odds ratio and corresponding 95% confidence intervals (CIs) for demographic, lifestyle, and comorbid factors related to chronic periodontitis (CP) concerning the development of rheumatoid arthritis (RA) when individuals are diagnosed with CP ≥ 2 within 1 year preceding the index date.

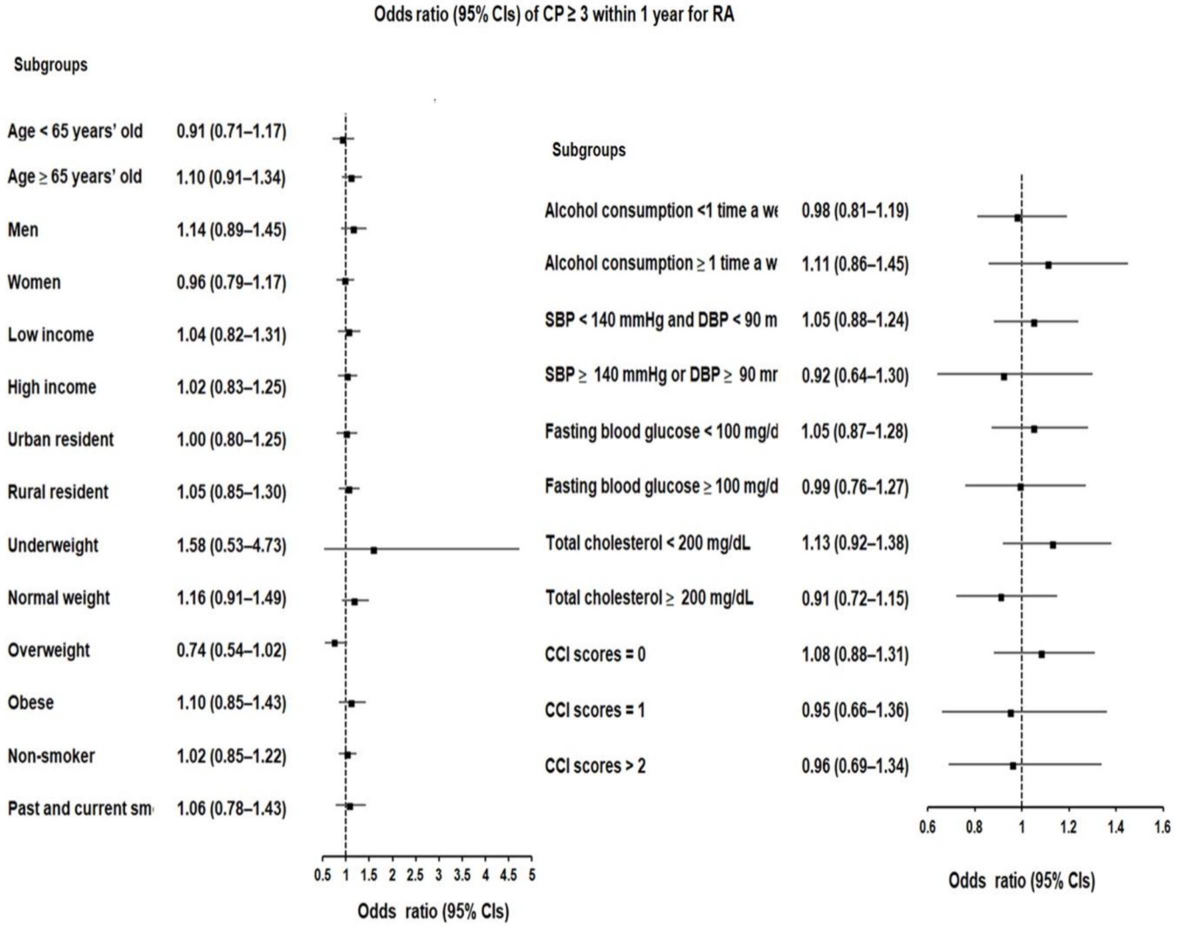

Supplementary Figure S3. Forest plots illustrating the adjusted odds ratio and corresponding 95% confidence intervals (CIs) for demographic, lifestyle, and comorbid factors related to chronic periodontitis (CP) concerning the development of rheumatoid arthritis (RA) when individuals are diagnosed with CP ≥ 3 within 1 year preceding the index date.
